# International Latin American Survey on Pediatric Intestinal Failure Team

**DOI:** 10.3390/nu13082754

**Published:** 2021-08-11

**Authors:** José Vicente N. Spolidoro, Mirella C. Souza, Helena A. S. Goldani, María N. Tanzi, Veronica B. Busoni, Maria del Carmen Padilla, Nelson E. Ramirez, Colomba Cofre, Lidia P. Valdivieso, Carola Saure, Gabriela Jimenez-Arguedas, Mikaelle S. M. Mateus, Roberta Serra, Carlos Cuadros-Mendonza, Juan Rivera-Medina, Daniela Gattini, Beatriz J. dos Santos, Clara Plata, Natascha Silva Sandy, on behalf of the LASPGHAN Intestinal Failure Working Group

**Affiliations:** 1 School of Medicine, Pontificia Universidade Catolica do Rio Grande do Sul, Porto Alegre 90619-900, Brazil; 2 Hospital Moinhos de Vento, Porto Alegre 90035-902, Brazil; 3 Unidade de Suporte de Home Care, Unimed Blumenau, Ponta Aguda 89051-900, Brazil; mirella.gastroped@gmail.com; 4 Pediatric Gastroenterology Unit, Hospital de Clinicas de Porto Alegre, Universidade Federal do Rio Grande do Sul, Porto Alegre 90035-903, Brazil; hegoldani@gmail.com; 5 Unidad de Nutrición Enteral y Parenteral, Pediatric Gastroenterology and Endoscopy, Centro Hospitalario Pereira Rossell, 11600 Montevideo, Uruguay; mntanzi@montevideo.com.uy; 6 Pediatric Gastroenterology and Hepatology Unit, Department of Pediatrics, Hospital Italiano de Buenos Aires, Buenos Aires C1199 CABA, Argentina; veronica.busoni@gmail.com; 7 Hospital Materno Infantil Caja Nacional, Santa Cruz, Bolivia; mari-al@hotmail.es; 8 Hospital del Seguro Universitário, Universidad Mayor de San Andrés, 3161 La Paz, Bolivia; nelramrod@gmail.com; 9 Department of Pediatric Gastroenterology and Nutrition, School of Medicine, Pontificia Universidad Catolica de Chile, Santiago 8330024, Chile; colombac@gmail.com (C.C.); dani.gattini@gmail.com (D.G.); 10 Pediatric Gastroenterology, Hepatology and Nutrition Unit, Department of Pediatrics, Hospital Nacional Docente Madre Niño San Bartolome, Lima 15001, Peru; patrilid23@yahoo.com; 11 División de Nutrición y Diabetes, Hospital de Pediatría Juan Pedro Garrahan, Buenos Aires C1245 CABA, Argentina; gocarola@icloud.com; 12 Servicio de Gastroenterología, Departamento de Pediatría, Hospital Nacional de Niños, “Dr. Carlos Sáenz Herrera” Caja Costarricense de Seguro Social, San José 10103, Costa Rica; gabyjimeneza@gmail.com; 13 Hospital Infantil Albert Sabin- Ambulatorio de Reabilitação Intestinal, Fortaleza 60410-994, Brazil; marquesmikaelle@gmail.com; 14 Instituto de Puericultura Martagã Gesteira—Universidade Federal do Rio de Janeiro, Rio de Janeiro 21941-912, Brazil; robertarcserra@gmail.com; 15 Hospital Internacional de Colombia—Fundacion Cardiovascular de Colombia, Santander 681004, Colombia; carloscuadros17@gmail.com; 16 Gastroenterology, Hepatology and Nutrition Unit of Instituto Nacional de Salud del Niño, Universidad Nacional Mayor de San Marcos, Lima 15081, Peru; juan.riveramedina@gmail.com; 17 Division of Gastroenterology, Hepatology and Nutrition, Department of Pediatrics, Hospital for Sick Children, University of Toronto, Toronto, ON M5G 1X8, Canada; natascha.sandy@hotmail.com; 18 Hospital da Criança Conceição, Porto Alegre 91350-250, Brazil; beajohn@terra.com.br; 19 Pontificia Universidad Javeriana, Hospital Universitario San Ignacio, Bogota 111321, Colombia; plata_clara@javeriana.edu.co

**Keywords:** home parenteral nutrition, intestinal failure, intestinal rehabilitation, survey

## Abstract

There is little data on the experience of managing pediatric Intestinal Failure (IF) in Latin America. This study aimed to identify and describe the current organization and practices of the IF teams in Latin America and the Caribbean. An online survey was sent to inquire about the existence of IF teams that managed children on home parenteral nutrition (HPN). Our questionnaire was based on a previously published European study with a similar goal. Twenty-four centers with pediatric IF teams in eight countries completed the survey, representing a total number of 316 children on HPN. The median number of children on parenteral nutrition (PN) at home per team was 5.5 (range 1–50). Teams consisted of the following members: pediatric gastroenterologist and a pediatric surgeon in all teams, dietician (95.8%), nurse (91.7%), social worker (79.2%), pharmacist (70.8%), oral therapist (62.5%), psychologist (58.3%), and physiotherapist (45.8%). The majority of the centers followed international standards of care on vascular access, parenteral and enteral nutrition, and IF medical and surgical management, but a significant percentage reported inability to monitor micronutrients, like vitamins A (37.5%), E (41.7%), B1 (66.7%), B2 (62.5%), B6 (62.5%), active B12 (58.3%); and trace elements—including zinc (29.2%), aluminum (75%), copper (37.5%), chromium (58.3%), selenium (58.3%), and manganese (58.3%). Conclusion: There is wide variation in how IF teams are structured in Latin America—while many countries have well-established Intestinal rehabilitation programs, a few do not follow international standards. Many countries did not report having an IF team managing pediatric patients on HPN.

## 1. Introduction

The cornerstone for the treatment of children with intestinal failure (IF) is specialized and individualized nutritional management, including a fine balance between parenteral nutrition (PN) and enteral nutrition (EN), with the ultimate goal to promote intestinal adaptation and enteral autonomy. Intestinal rehabilitation (IR) is typically a long process in which different phases of nutritional management can be recognized. After an initial acute phase which focuses on metabolic stabilization, the adaptative period starts, and during this phase, many patients are candidates for home parenteral nutrition (HPN) [1]. There is extensive evidence to endorse the recommendation that children with IF are best managed by an Intestinal rehabilitation program (IRP) [2], as delivery of care by these programs has been associated with significantly reduced morbidity and mortality in this chronic complex condition [3]. For more than three decades, IRPs exist in multiple sites across the globe, especially in North America and Europe, and management of IF by IRPs is considered the current state of the art [2].

In 2017, the Nutrition Committee of the North American Society for Pediatric Gastroenterology, Hepatology and Nutrition (NASPGHAN) released a Society paper highlighting the importance of IRP in the management of IF and Short Bowel Syndrome (SBS), summarizing the IRP experience of numerous programs, networks, and consortiums [2]. NASPGHAN recommended that at minimum staffing for an IR program should include a gastroenterologist, surgeon, dietitian (or registered dietitian-nutritionist), and a nurse, although the need for close collaboration with neonatologists, and the role for many other specialists and health care professionals (social workers, child psychologists, occupational therapists/physical therapists, speech/feeding therapists, interventional radiologists, and child-life specialists) was also recognized.

In that same year, a European study on the organization and clinical practice of teams treating children with IF across Europe revealed a wide diversity of composition of these teams and their number of patients treated [4]. Furthermore, these authors assessed compliance of the IF teams with the most current guideline at the time of their publication provided by the European Society for Paediatric Gastroenterology, Hepatology and Nutrition (ESPGHAN) and the European Society for Clinical Nutrition and Metabolism (ESPEN) [5].

To date, despite all that significant progress in understanding the importance of establishing these programs, the need for quality assurance and research collaboration among the programs, there is relatively scarce data published on the experience managing pediatric IF in Latin America. In 2018, Gondolesi et al. [6] summarized the data using existing publications and personal surveys to provide a preliminary overview on the management of intestinal failure in middle-income countries from Latin America and Asia (children and adults) [6]. The authors reported that HPN is still not available in all Latin American countries, and that there was significant disparity among these countries, highlighting the need to pursue the development of registries, guidelines, and health policies.

Our study aimed to identify the Intestinal Rehabilitation Centers for children with IF in Latin America and the Caribbean led by members of the Latin-American Society for Pediatric Gastroenterology and Nutrition (LASPGHAN), and to provide an overview of the current organization and practices of specialized pediatric IF teams.

## 2. Materials and Methods

### 2.1. Study Design

Our questionnaire was heavily based on the previously published European international survey questionnaire, which consisted of 68 questions regarding local protocols and strategies [4]. After contacting the authors of that study, and making adjustments to the questionnaire to our local practices and reality in Latin America, the survey was translated into Portuguese and Spanish. Among the adaptations, we did not include questions about lipid and protein target administration according to ESPEN/ESPGHAN Guidelines.

We conducted an online survey between August 2020 and April 2021.

### 2.2. Participants and Recruitment

An invitation to the survey was electronically sent to all members of LASPGHAN. In addition, respondents were asked to forward the survey to any other IRPs or IF teams in their country, as well as any colleagues they knew to be involved in the management of children with IF. As there is no register of centers providing HPN in Latin America, and we also asked for and expected a “snowball” recruitment, in order to enroll as many IRPs and IF centers as possible, it is not known exactly how many teams providing care for children with IF were invited to complete our survey. However, as several messages were sent to all leaders of pediatric gastroenterology centers in Latin America, and in social media groups involved with IF. The idea of this survey is to have a picture of the current situation of IF in Latin America.

The inclusion criteria were centers that had pediatric patients with IF (age 0–18 years), currently on HPN.

### 2.3. Data Collected

Overall participants were asked data on seven broader domains: (1) General information on place of work, IF teams and patients managed—institution and country of work, experience of the respondent and the IF team managing children on HPN, composition of the IF team, number and age of patients, underlying causes of IF, and whether the team continue to monitor patients weaned off HPN; (2) Vascular access—type of central line used for HPN, use of lock solutions in central lines, anticoagulation to prevent catheter-related thrombosis, and management of central line occlusion; (3) HPN solutions and administration—type of HPN provided and source of funding for HPN, practices around use of lipid emulsion and lipid strategies in case of Intestinal Failure Associated Liver Disease (IFALD), and person in charge of HPN administration (parents/caregivers vs. home-care nursing); (4) Enteral and Oral nutrition—use of breastmilk and formulas for newborn and infant with SBS, type of formula feeds used for older children with SBS, delivery of EN, usual period of time for the removal of central line once full feeds are achieved, and involvement of oral therapist for oral nutrition; (5) Nutritional status and bone health monitoring—clinical and laboratory measurements used to assess nutritional status and requirements and type of measurements used and frequency; (6) Medical and surgical management of IF—medications used in the management of intestinal failure, type and frequency of nontransplant (bowel lengthening procedures for SBS), and transplant surgical procedures performed; and (7) Neuropsychological and psychomotor development—attendance to school and special needs, neuropsychological, and psychomotor assessments. The questionnaire is available as a supplement (Appendix A).

### 2.4. Data Summary

Descriptive statistics were used to analyze responses using Excel (Microsoft, Redmond, WA) and Google Surveys software. Categorical variables were summarized as frequencies and percentages, and continuous variables as median and interquartile range (IQR).

## 3. Results

### 3.1. General Information on Place of Work, IF Teams and Patients Managed

**Figure 1:**
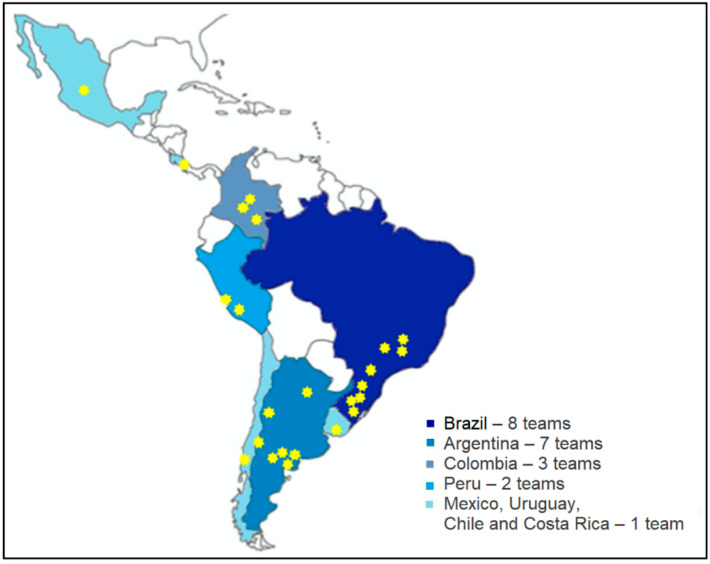
General information on place of work.

Twenty-three centers in seven countries responded to the survey. Brazil and Argentina were the countries with a higher number of centers. The distribution of centers among the different countries is summarized in Figure 1. None of the questionnaires were removed because of missing data. Given that LASPGHAN has contacted Gastroenterology, Hepatology, and Pediatric Nutrition centers in all countries in Latin America, we believe that all or almost the totality of the rehabilitation programs in the region were represented in the present study. Fifty percent of these programs are in pediatric hospitals, and 58.3% are university-affiliated hospitals.

Seventy-eight percent of the IRPs teams are coordinated by physicians specialized in gastroenterology or pediatric nutrition. A third of the programs have been caring for children with IF on HPN for more than a decade (three of these programs for more than two decades). The experience of the IF teams in Latin America is presented and compared to the European experience in Table 1. In terms of the composition of the IF teams and the minimum staffing according to NASPGHAN recommendations [2], all teams included pediatric gastroenterologists and pediatric surgeons, all but one (95.8%) included a dedicated dietician, and all but two (91.7%) included a nurse. These data and the presence of other important heath care professional who participate in IRP is summarized in Table 2. The composition of the teams was relatively similar to that described in the European study [4] (Table 2).

At the time of the survey, a total of 316 children were being cared for as outpatients with HPN. The number of children managed by each team varied from 1 to 50, with a median of 5.5 (interquartile range 3–20): five centers with more than 30 patients (two in Brazil and three in Argentina), while eleven centers managed five or fewer patients. Eighty percent of the patients have SBS, 14% dysmotility, and 5.4% enteropathy. Age distribution of patients on HPN is summarized in Table 3. All centers but one continued to monitor patients who were on HPN after weaning off PN. Only 25% offered a structured transition to adult care for children with IF.

### 3.2. Vascular Access

All teams reported using tunneled central venous catheters (CVC), such as Broviac^®^ or Hickman^®^ catheters as their first choice. Solutions to prevent central line-associated bloodstream infection (CLABSIs) were reported by 58.3% of the respondents—33.3% used taurolidine lock; 25% used ethanol lock. The only reason reported for the lack of use of taurolidine was lack of availability, while for not using ethanol, the justifications were divided between the consideration that the alternative (taurolidine) was perceived as more effective, and lack of availability. None of the respondents reported a lack of knowledge about the existence/use of these two lock therapies. For the prevention of catheter-related thrombosis/occlusion, 58.3% of the teams reported using anticoagulation, and the standard therapy was low-molecular-weight heparin (LMWH) in all cases. Finally, for the management of catheter occlusion, 39.1% reported the use of alteplase, 30.4% use heparin, and 13% use of urokinase.

### 3.3. HPN Solutions and Administration

In terms of how HPN solution is primarily provided, 75% reported that a customized HPN solution is prepared by a specialized pharmacy (not affiliated to a hospital), 33.3% customized HPN prepared by a hospital pharmacy, and two teams occasionally used standardized commercially available PN. Costs of HPN are covered solely by the public health care system in 41.6% of the programs, while 33.3% of teams worked with HPN covered solely by private insurances. The remaining programs provided HPN covered either by hospitals, and/or public health care system, and/or private insurance. Interestingly, caregivers/parents were in charge of HPN administration in half the cases (50%), while in the other half (50%) a home care team provided this care. When parents were in charge of PN administration the usual period of training to deliver this care was between 2 to 4 weeks.

Soybean/MCT/olive/fish oil (SMOF lipid^®^) emulsion was the first choice of lipid emulsion reported by all but two centers—one reported using a mixed soybean oil and medium-chain triglycerides emulsion (Lipofundin^®^) and one using soybean oil lipid emulsion (Intralipid^®^).

### 3.4. Enteral and Oral Nutrition

When feeds were introduced in neonates/infants’ diet, all teams recommended breast milk as the first choice, and when breast milk was not available for these infants, extensively hydrolyzed formula was the preferred initial choice by 79.2% of the programs, intact protein formula by 12.5% and amino acid-based formula by 8.3%. For older children, the recommended type of feeding at the time diet was initiated was extensively hydrolyzed pediatric formula by 54.2% of the IF teams, intact protein pediatric formula by 33.3%, and solid oral feeding by 12.5%.

In terms of feeding delivery, 45.8% of the programs reported that the most commonly used was a combination of intermittent and continuous feeding, 37.5% oral feeds, 8.3% intermittent tube feeds, and 8.3% continuous tube feeds. Seventy-nine percent of teams have an oral therapist routinely participating in the introduction of oral feeds.

### 3.5. Nutritional Status and Bone Health Monitoring

For standard monitoring, routine measurements of weight, height, and body mass index (BMI) were universally used (100%); head circumference by 87.5%, mid-upper arm circumference (MUAC) by 62.5%, and skinfolds by 37.5% of the teams. Blood work was regularly done by all centers as part of nutritional monitoring, but many parameters that were normally monitored as part of periodic follow-up in developed countries, were not followed in some programs. A significant percentage of programs reported inability to monitor vitamin A, vitamin E, vitamin B1, vitamin B2, vitamin B6, active vitamin B12, zinc, aluminum, copper, chromium, selenium, and manganese (Table 4). For bone health monitoring, blood parameters (including calcium, phosphate, and vitamin D) were used by all teams (in 95.8% at least every 6 months), whereas Dual-energy X-ray absorptiometry (DXA) was performed at least yearly by 50% of teams.

### 3.6. Medical and Surgical Management of IF

More than 90% of teams regularly use a proton pump inhibitor (PPI), loperamide, and antibiotics for small intestine bacterial overgrowth (SIBO); while more than half used bile acid sequestrants and prokinetics, and more than a third used probiotics. The use of these medications and other medical therapies is summarized on Table 5.

Bowel lengthening procedures for SBS were offered by 91.7% of teams, all of these were able to offer Serial transverse enteroplasty (STEP) procedures, and Bianchi procedures by 8.3%. The number of STEP procedures performed annually varied from 1 to 10. Small bowel transplant is offered by three services (16.7%), one in Brazil and two in Argentina.

### 3.7. Neuropsychological and Psychomotor Development

Neuropsychological and psychomotor development standard assessments were reported by 70.8% of centers. According to 58.3% of the IF teams, most of the children independently attended to regular schools, while 20.8% reported that the majority attended regular schools but with extra assistance.

## 4. Discussion

Intestinal failure is a knowingly life-threatening medical condition, which demands complex, interdisciplinary, and costly medical care that ideally should follow similar international standards to allow patients with this severe condition to have optimal outcomes. However, in reality, we know there are many barriers to that—to name a few: the relative rarity of this entity, the high cost of treatment, the need for centralization in care, and the disparity of medical care provided among different regions. Our Latin American and Caribbean survey showed a significant disparity around the care available for children with IF in the area. The region comprehends 20 countries; however, our survey identified 24 teams with children on HPN at the time of this survey, distributed among eight countries. This survey could not find any country with IRPs with HPN children in the Caribbean Region. Other centers can take care of IF children, but they did not have patients on HPN during this survey period and they were not included in this study. In addition, within those 24 teams, we observed that 22 (91.7%) were composed of the recommended minimum staffing for a pediatric IR program according to NASPGHAN endorsement [2]—including at least a gastroenterologist, surgeon, dietitian, and a nurse. We noted that a smaller percentage of the centers reported a social worker (79.2%), a pharmacist (70.8%), and a psychologist (58.3%) as part of the team. These team members, although recognized as an important part of an IRP, are not among the minimum team recommended by NASPGHAN [2], but are mentioned by ESPGHAN/ESPEN guidelines on pediatric PN as part of the multidisciplinary nutrition support team [4,7,8]. In the international European survey, Neelis. et al. reported this large diversity in the composition and organization of pediatric IF teams, with only 46% of teams following their local guidelines and composed by a physician, pharmacist, nurse, dietician, social worker, and psychologist [4]. The composition of Latin American and European teams was quite similar (Table 2).

Beyond the variability in the composition of the teams, again in keeping with findings by Neelis et al. [4] and previously reported in the United Kingdom [9,10], we found a large variation in the number of patients cared for by each team, but our median number (5.5) was lower than the number reported in the European survey (median = 15). Additionally, we noted a disparity in the experience of the teams: while a third existed for more than a decade, 37.5% have been managing children on HPN for less than 5 years (Table 1).

Overall, the practices reported around vascular access and management (including use of a lock solution to prevent CLABSIs, anticoagulation, and approach to CVC occlusion) and oral and enteral nutrition were relatively uniform. There was some variation in practices related to the availability of resources, local practices and regulations, and some variability in areas with the weakest evidence. For instance, in our study just over half of the teams (58.3%) reported using anticoagulation as primary prophylaxis in the prevention of catheter-related thrombosis—this number was comparable to the percentage reported in Europe by Neelis et al. [4], and interesting that when these authors performed a sub-analysis comparing smaller (teams with ≤10 patients on HPN) and larger centers (teams with >10 patients on HPN), they found that prophylactic anticoagulation was used significantly more frequently in the teams with more patients (59% vs. 28%)—of note, a consensus has not been reached to recommend prophylactic anticoagulation universally in children with IF. On the other hand, the use of SMOF lipid^®^ emulsion was the first choice reported by more than 90% of the teams—this emulsion was shown to significantly reduce the risk of progressive IFALD in children with IF [11], and largely adopted as the lipid of choice for children with IF for the treatment and prevention of IFALD [12]. These two examples illustrate in a way the reality of managing IF: more uniform practices in areas where the evidence is stronger (e.g., lipid of choice) and significant variability where there is a lack of recommendations for daily practice (e.g., primary anticoagulation).

In terms of feeding strategies, probably the recommendation with the most agreement among experts is that in infants with IF, human milk is considered to be the first choice [1,13]—beyond the amply documented health benefits of human milk, it is hypothesized that different components of breast milk may themselves potentialize intestinal adaptation (such as glutamine, growth hormone, and epidermal growth factor), while other components (such as nucleotides, immunoglobulin A, leucocytes, immunoglobulins, and antimicrobial peptides) provide important immunological support and promote a favorable microbiome environment [14]. Fortunately, we found that breast milk was the universal first choice reported when feeds were introduced in neonates/infants’ diet.

Notably, our reported practices on regular monitoring of bone health and micronutrient status are far from desirable and the practices reported in Europe and North America: we reported an inability to monitor vitamins (A, E, B1, B2, B6, and B12) and micronutrients (zinc, aluminum, copper, chromium, selenium, and manganese) that are knowingly important in the context of IF. PN dependence was consistently reported by the teams with the absence of monitoring by 37.5 to 75%, depending on the micronutrient in question, while DXA was monitored yearly by 50%.

Neelis et al. [4] highlighted the importance of being attentive to these patients’ neuropsychological and psychomotor development, and in our survey we found, likewise, that standard assessments were reported by just over two thirds of centers (70.8%), and that most of the children independently attend regular schools, with a smaller percentage requiring extra assistance.

Our study limitations included the well-known limitations of survey-based studies. Furthermore, we could not calculate a response rate given the methodology of choice sending out the surveys—just like it happened in the pioneer European survey [4], this impossibility of calculating a response rate results from the lack of clarity or previous knowledge of which Latin American centers have a pediatric IF team and received the online survey. We have sent an invitation to the survey via email using the LASPGHAN platform (with a historical archive on contact information of LASPGHAN’s current members and inactive members) and made the survey available in the LASPGHAN website. Additionally, also received some responses from other teams not directly invited by us. Therefore, it is unknown how many teams did not participate. Nevertheless, given that the leadership of LASPGHAN is quite diverse and that the field of IF is a relatively small community, we believe it represented the IF teams in near totality. However, we cannot present a response rate for our survey for all the reasons above-mentioned.

## 5. Conclusions

In conclusion, our study is the first to assess in details the care by the pediatric Intestinal Rehabilitation Programs taking care of HPN children in Latin America and the Caribbean, where we found that the care delivered follows international standards in many aspects; however, many countries in this region do not have these programs established. Furthermore, it seems that the area in which our practices lag behind the care offered in Europe and North America is on regular monitoring of bone health and micronutrient status. We hope that a process of quality improvement will be put in place to improve compliance with international standards on those aspects. Finally, we conclude that, as reported by our European colleagues [4], there seems to be a wide diversity in the organization of these programs and among some aspects of clinical practice delivered by these teams.

## Figures and Tables

**Table 1:** Experience of the IF teams in Latin America and Europe [4].

Experience of the IF Teams	Latin America *n* (%)	Europe (%)
≤1 year	1 (4.2)	2
>1 to ≤5 years	8 (33.3)	5
>5 to ≤10 years	7 (29.2)	15
>10 to ≤20 years	4 (20.8)	49
>20 years	3 (12.5)	30

**Table 2:** Composition of IF teams in Latin America and Europe [4].

Professional	Latin America *n* (%)	Europe (%)
Pediatric Gastroenterologist	24 (100)	100
Pediatric surgeon	24 (100)	89
Dietician	23 (95.8)	95
Nurse	22 (91.7)	92
Social Worker	19 (79.2)	62
Pharmacist	17 (70.8)	82
Speech therapist	15 (62.5)	48
Psychologist	14 (58.3)	66
Physiotherapist	11 (45.8)	38
General pediatrician	02 (8.3)	33
Pediatric intensivist, interventional radiologist	1 (4.2)	NR
Child life Specialist, Occupational therapist	1 (4.2)	NR

NR = Not Reported.

**Table 3:** Demographic distribution by age groups in Latin America and Europe [4].

		Latin America	Europe
Patients age distribution	*n* (%)	Median (IQR;min–max)	Median (IQR;min–max)
Younger than 1 year	25 (7.9)	1 (0–1; 1–5)	1 (1–3; 0–25)
1 to 5 years	178 (56.3)	3 (1–9; 0–43)	5 (2–9; 0–45)
5 to 10 years	66 (20.9)	1.5 (0–3; 0–15)	3 (2–5; 0–34)
10 to 15 years	35 (11.1)	0.5 (0–2; 0–10)	2 (1–5; 0–30)
Older than 15 years	12 (3.8)	0 (0–1; 0–3)	1 (0–5; 0–23)

**Table 4:** Frequency of micronutrient monitoring in Latin America teams.

Micronutrient	Every 3 Months	Every 6 Months	Yearly	Every 2 Years	Never	Unknown
25-OH vitamin D	62.5%	33.3%	4.2%	-	-	-
Vitamin A	16.7%	33.3%	8.3%	4.2%	37.5%	-
Vitamin E	16.7%	29.2%	4.1%	4.1%	41.8%	4.1%
Vitamin B1	16.7%	8.3%	4.2%	-	66.7%	4.1%
Vitamin B2	16.7%	12.5%	8.3%	-	62.5%	-
Vitamin B6	16.7%	12.5%	8.3%	-	62.5%	-
Vitamin B12 active	12.5%	25%	-	-	58.3%	4.2%
Vitamin B12	29.2%	62.5%	8.3%	-	-	
Zinc	33.3%	25%%	8.3%	4.2%	29.2%	
Aluminum	8.3%	16.7%	-	-	75%	-
Cooper	16.7%	25%	16.7%	4.3%	37.5%	-
Chromium	16.7%	16.7%	8.3%	-	58.3%	-
Selenium	12.5%%	25%	4.2%	-	58.3%	-
Manganese	16.7%	16.7%	4.2%	-	56.5%	4.1%

**Table 5:** Medications regularly used by the IF teams in Latin America and Europe [4].

	Latin America	Europe
Medication	Number of IF teams (%)	Number of IF teams (%)
Antibiotics as treatment for small intestinal bacterial overgrowth (e.g., metronidazole)	22 (91.7)	53 (90)
Proton pomp inhibitor (e.g., omeprazole)	23 (95.7)	53 (90)
Antidiarrheal/antimotility agents (e.g., loperamide)	22 (91.7)	43 (73)
Bile acid sequestrant (e.g., cholestyramine)	15 (62.5)	38 (64)
Histamine receptor antagonist (e.g., ranitidine)	05 (20.8)	32 (54)
Probiotics	08 (33.3)	26 (44)
Prokinetic agents (e.g., erythromycin)	12 (50)	22 (37)
Somastostatin analogue (e.g., octreotide)	05 (20.8)	8 (14)
Antisecretory (e.g., racecadotril)	03 (12.5)	?
A2-adrenergic receptor agonist (e.g., clonidine)	01 (4.2)	2 (3)
Growth factors (e.g., Glucagon-like peptide-2 analogue-teduglutide)	02 (8.3)	2 (3)

## Data Availability

All data presented in this study, not yet publicly archived, shall be made available through the corresponding author on request.

## Appendix A

     **QUESTIONNAIRE FOR TEAMS OF INTESTINAL REHABILITATION PEDIATRICS OF LATIN AMERICA**

**Your participation is critical.**

**We want to know how our INTESTINE REHABILITATION teams are working**

**If you know of other centers that work with INTESTINE REHABILITATION in Latin America, please forward the link sent to you**

***Mandatory**

1. Email *

2. What is your country of work? *

3. What is your city of work? *

4. What is the name of your institution? *

5. What type of hospital do you work for? * Mark only one option.

   General Hospital

   General University Hospital Pediatric Hospital

   University Pediatric Hospital

6. What is your profession? * Mark only one option.

   Pediatric Gastroenterologist/Nutrologist

   Pediatric Surgeon

   General Pediatrician

   Nutritionist

   Nurse

   Other:

7. How long has your Intestinal Failure team been following children on home parenteral nutrition (HPN)? *

   <1 year

   1– years

   6–10 years

   11–20 years

   >20 years

8. How many years of experience do you have working on a Intestinal Bankruptcy team? *

   <1 year

   1–5 years

   6–10 years

   11–20 years

   >20 years

9. Does your Pediatric Bowel Bankruptcy team articulate with an adult Bowel Bankruptcy team? Do you offer a transition service for Bowel Failure patients? *

   YES

   NO

10. Who are the professionals involved in your Intestinal Rehabilitation Center? Multiple choice *

   Pediatric Gastroenterologist/Physician who specializes in Nutrition

   Pediatric Surgeon

   Nutritionist

   Nurse

   Pharmacist

   Social Worker

   Speech-Language Pathologist

   Physiotherapist

   Psychologist

   Other:

11. In any of the professions selected above, is there more than 1 member in your Intestinal Rehabilitation Center? Write which and how many members of this profession are part of it. *

12. What is the current number of children (<18 years old) in HPN who are on your Bowel Failure team? *

**All requested information refers to patients currently receiving home parenteral nutrition**

**The following 3 questions address the distribution of underlying causes of Bowel Failure.**

13. How many children have short bowel syndrome? *

14. How many children have a motility disorder? *

15. How many children have enteropathy? *

**The 5 questions below refer to the age of patients in Home Parenteral Nutrition**

16. How many are under 1 year old? *

17. How many are 1 to 5 years old?*

18. How many are between 5 and 10 years old? *

19. How many are 10 to 15 years old? *

20. How many are > 15 years old? *

21.Does your Intestinal team continue to monitor children who have become independent HPN? *

   Yes

   No

**VASCULAR ACCESS**

22. What type of central venous catheter do you prefer to use for HPN administration? *

Central Venous Catheter—Tunneled (e.g., Broviac^®^ or Hickmann^®^)

Central Venous Catheter: Peripheral Inserted Non-Tunneled Central Catheter (PICC)

   Port-a-cath

   Other:

23. What catheter lock solution does your center use to prevent catheter occlusion? *

Mark only one option.

   Heparin

   Saline solution

   I don’t use any

24. What catheter lock solution does your center use to prevent catheter infection? *

Mark only one option.

   Ethanol

   Taurosept^®^ (taurolidine)

   TaurolockTM (taurolidine and citrate)

   I don’t use any

25. In case of not using ethanol block, explain the reason: * Mark only one option.

lack of availability

   High cost

   I think that what I use is more effective

   I don’t know this option

   I use ethanol

26. In case of not using taurolidine block, explain why: * Mark only one option.

lack of availability

   High cost

   I think that what I use is more effective

   I don’t know this option

   I use taurolidine

27. Do you use anticoagulation to prevent catheter-related deep vein thrombosis? *

Mark only one option.

   Yes

   No

28. Yes: What type of anticoagulation? Mark only one option.

   Low molecular weight heparin (e.g., nadroparin) subcutaneously

   Vitamin K antagonists (e.g., acenocoumarol)

   Other:

29. Do you have a standard procedure for catheter occlusion? * Mark only one option.

   Yes

   sometimes

   never

30. YES: What is used in case of catheter occlusion? Mark only one option.

   Blockade of Heparin

   Urokinase

   Alteplase

   Other:

**HOME PARENTERAL NUTRITION SOLUTIONS**

31. How is HPN provided at your institution? Multiple choice * Check all that apply.

Personalized, prepared by your Hospital’s Pharmacy

Personalized, prepared by an out-of-hospital specialized pharmacy

Commercial mixed bags with infusion of vitamins and trace elements outside the parenteral bag

Commercial mixed pouches manipulated at the Pharmacy to add vitamins and trace elements

Commercial mixed pouches manipulated at home to add vitamins and trace elements

Commercial mixed bags manipulated at home to add vitamins and trace elements

Other:

32. Which lipid emulsion is preferentially used for HPN? * Mark only one option.

   Soy Lipid Emulsion (e.g., Intralipid^®^)

   Soy Lipid Emulsion/Medium Chain Triglycerides (MCT)/Olive/Fish Oil (e.g., SMOFlipid^®^)

   Fish Oil Lipid Emulsion (e.g., Omegaven^®^)

   Olive/Soy Lipid Emulsion (e.g., Clinoleic^®^)

   Soy/TCM Lipid Emulsion (e.g., Lipofundin^®^)

   Other:

33. If you have moderate to severe liver disease associated with intestinal failure, do you reduce the days of the week you infuse lipids? *

Mark only one option.

   Yes

   No

34. How is HPN funded? Multiple choice * Check all that apply.

   private health insurance

   public health system

   hospital

   I do not know

   Other:

35. In general, who administers the HPN? * Mark only one option.

   Parents/caregivers

   Home Care Companies

36. If parents/caregivers: Who trains parents/caregivers to administer HPN and care for the central venous catheter? And how long does this training take? *

**ENTERAL/ORAL NUTRITION**

37. Does your intestinal rehabilitation team start a breast milk diet for newborns with a short bowel? *

Mark only one option.

   YES

   NO

38. What type of feeding is recommended preferentially when starting the nutrition of newborns/babies if there is no breast milk? *

Mark only one option.

- Polymeric (containing whole proteins, complex carbohydrates and TCL)

- Oligomeric (which contains hydrolyzed proteins, complex carbohydrates and TCM)

- Monomeric (containing amino acids, complex carbohydrates and TCL)

39. What type of diet is best recommended when starting nutrition in older children? *

Mark only one option.

   Polymeric (containing whole proteins, complex carbohydrates and TCL) Oligomeric (containing hydrolyzed proteins, complex carbohydrates and TCM)

   Monomeric (containing amino acids, complex carbohydrates and TCL) Solid oral feeding

40. How is enteral nutrition most often administered? * Mark only one option.

   Intermittent/bolus feeding (e.g., 6 times of 120 mL) orally

   Intermittent/bolus feeding (e.g., 6 times of 120 mL)—per tube

   Continuous (e.g., continuous tube feeding of 30 mL per hour, 24 h)

   Combination of intermittent and continuous feeding (e.g., intermittent during the day and continuous at night)

41. After achieving complete enteral nutrition, how long after, on average, is the central venous catheter removed (ie after how many days/weeks/months)? *

42. Does a SPEECH THERAPIST participate in the introduction of oral feeding? * Mark only one option.

   YES

   NO

**MONITORING OF NUTRITIONAL STATUS**

43. How is nutritional status monitored regularly? Multiple choice * Check all that apply.

   Weight

   Height

   Cephalic perimeter

   BMI

   Arm/calf circumference

   Skin fold

   Densitometry

   Air Displacement Plethysmography

   Other:

45. How often are vitamin A levels usually measured? * Mark only one option.

   Never

   Every 3 months

   Every 6 months

   Annually

   Every 2 years Interval

   >2 years

   I don’t know

46. How often are vitamin E levels usually measured? * Mark only one option.

   Never

   Every 3 months

   Every 6 months

   Annually

   Every 2 years Interval

   >2 years

   I don’t know

47. How often are vitamin B1 levels usually measured? * Mark only one option.

   Never

   Every 3 months

   Every 6 months

   Annually

   Every 2 years Interval

   >2 years

   I don’t know

48. How often are vitamin B2 levels usually measured? * Mark only one option.

   Never

   Every 3 months

   Every 6 months

   Annually

   Every 2 years Interval

   >2 years

   I don’t know

49. How often are vitamin B6 levels usually measured? * Mark only one option.

   Never

   Every 3 months

   Every 6 months

   Annually

   Every 2 years Interval

   >2 years

   I don’t know

50. How often are active vitamin B12 levels usually measured? * Mark only one option.

   Never

   Every 3 months

   Every 6 months

   Annually

   Every 2 years Interval

   >2 years

   I don’t know

51. How often are vitamin B12 levels usually measured? * Mark only one option.

   Never

   Every 3 months

   Every 6 months

   Annually

   Every 2 years Interval

   >2 years

   I don’t know

52. How often are Zinc levels usually measured? * Mark only one option.

   Never

   Every 3 months

   Every 6 months

   Annually

   Every 2 years Interval

   >2 years

   I don’t know

53. How often are Aluminum levels usually measured? * Mark only one option.

   Never

   Every 3 months

   Every 6 months

   Annually

   Every 2 years Interval

   >2 years

   I don’t know

54. How often are copper levels usually measured? * Mark only one option.

   Never

   Every 3 months

   Every 6 months

   Annually

   Every 2 years Interval

   >2 years

   I don’t know

55. How often are Chromium levels usually measured? * Mark only one option.

   Never

   Every 3 months

   Every 6 months

   Annually

   Every 2 years Interval

   >2 years

   I don’t know

56. How often are Selenium levels usually measured? * Mark only one option.

   Never

   Every 3 months

   Every 6 months

   Annually

   Every 2 years Interval

   >2 years

   I don’t know

57.How often are Manganese levels usually measured? * Mark only one option.

   Never

   Every 3 months

   Every 6 months

   Annually

   Every 2 years Interval

   >2 years

   I don’t know

**BONE HEALTH MONITORING**

58. How is bone health monitored in children with Bowel Failure? And how often does your team monitor blood parameters (e.g., calcium, phosphate, vitamin D)? *

Mark only one option.

   Never

   Every 3 months

   Every 6 months

   Annually

   Every 2 years Interval

   >2 years

   I don’t know

59.How often is bone health monitored in children with Bowel Failure by Bone Densitometry? *

Mark only one option.

   Never

   Every 3 months

   Every 6 months

   Annually

   Every 2 years Interval

   >2 years

   I don’t know

60. How often is bone health monitored in children with Bowel Failure by X-RAYS (radiography of the hand using BoneXpert software)? *

Mark only one option.

   Never

   Every 3 months

   Every 6 months

   Annually

   Every 2 years Interval

   >2 years

   I don’t know

**SURGERY/MEDICINES**

61. Which of the following procedures are performed in your Center? Multiple choice *

Check all that apply.

   Serial Transverse Enteroplasty (STEP)

   Bianchi Procedure

   Intestinal transplant

   None of these procedures

62. How often is serial transverse enteroplasty (STEP) performed? Number of procedures/year *

63. How often is the BIANCHI PROCEDURE (LILT) performed? Number of procedures/year *

64. How often is INTESTINE TRANSPLANTATION performed? Number of procedures/year *

65. Which of the following medications do you regularly use for children who use HPN? Multiple choice *

   Check all that apply.

   Histamine receptor antagonist (e.g., Ranitidine)

   Proton pump inhibitor (e.g., omeprazole)

   2-Adrenergic Receptor Agonist (e.g., clonidine)

   Somatostatin analogue (e.g., octreotide)

   Anti-diarrheal/anti-motility agents (e.g., loperamide)

   Bile acid chelators (e.g., Cholestyramine)

   Prokinetic agents (e.g., erythromycin)

   Antibiotics as a treatment for small bowel bacterial overgrowth (e.g., metronidazole)

   Antisecretory agents (e.g., racecadotril)

   Probiotics

   Growth factors (e.g., GLP-2 analogue teduglutide)

   Other:

**NEUROPSYCHOLOGICAL AND PSYCHOMOTOR DEVELOPMENT/GENERAL ISSUES**

66. In general, what is the intellectual performance of children treated with HPN by your staff when they go to primary school? *

Mark only one option.

   Most of them attend regular schools

   Most of them attend regular schools with additional help.

   Most of them attend schools for children with special needs (for medical reasons)

   Most of them attend schools for children with special needs (for intellectual reasons)

   Other:

67. If you like, write a comment in the previous question

68. Is neuropsychological and psychomotor development regularly assessed in your Intestinal Rehabilitation Center? *

Mark only one option.

   YES

   NO

**FINAL IMPRESSIONS**

69. Do you have anything to add to this survey?

## References

[1] Goulet O., Olieman J., Ksiazyk J., Spolidoro J., Tibboe D., Köhler H., Yagci R.V., Falconer J., Grimble G., Beattie R.M. (2013). Neonatal short bowel syndrome as a model of intestinal failure: Physiological background for enteral feeding. Clin. Nutr..

[2] Merritt R.J., Cohran V., Raphael B.P., Sentongo T., Volpert D., Warner B.W., Goday P.S. (2017). Intestinal Rehabilitation Programs in the Management of Pediatric Intestinal Failure and Short Bowel Syndrome. J. Pediatr. Gastroenterol. Nutr..

[3] Stanger J.D., Oliveira C., Blackmore C., Avitzur Y., Wales P.W. (2013). The impact of multi-disciplinary intestinal rehabilitation programs on the outcome of pediatric patients with intestinal failure: A systematic review and meta-analysis. J. Pediatr. Surg..

[4] Neelis E., De Koning B., Van Winckel M., Tabbers M., Hill S., Hulst J. (2018). Wide variation in organisation and clinical practice of paediatric intestinal failure teams: An international survey. Clin. Nutr..

[5] Jones N.L., Koletzko S., Goodman K., Bontems P., Cadranel S., Casswall T., Czinn S., Gold B.D., Guarner J., Elitsur Y. (2017). Joint ESPGHAN/NASPGHAN Guidelines for the Management of Helicobacter pylori in Children and Adolescents (Update 2016). J. Pediatr. Gastroenterol. Nutr..

[6] Gondolesi G.E., Pattín F., Nikoupour H. (2018). Management of intestinal failure in middle-income countries, for children and adults. Curr. Opin. Organ Transplant..

[7] Puntis J., Hojsak I., Ksiazyk J., Braegger C., Bronsky J., Cai W., Campoy C., Carnielli V., Darmaun D., Decsi T. (2018). ESPGHAN/ESPEN/ESPR/CSPEN guidelines on pediatric parenteral nutrition: Organisational aspects. Clin. Nutr..

[8] Koletzko B., Goulet O., Hunt J., Krohn K., Shamir R. (2005). Guidelines on Paediatric Parenteral Nutrition of the European Society of Paediatric Gastroenterology, Hepatology and Nutrition (ESPGHAN) and the European Society for Clinical Nutrition and Metabolism (ESPEN), Supported by the European Society of Paediatric Research (ESPR). J. Pediatr. Gastroenterol. Nutr..

[9] Koglmeier J., Day C., Puntis J.W.L. (2008). Clinical outcome in patients from a single region who were dependent on parenteral nutrition for 28 days or more. Arch. Dis. Child..

[10] Beath S., Gowen H., Puntis J. (2011). Trends in paediatric home parenteral nutrition and implications for service development. Clin. Nutr..

[11] Diamond I.R., Grant R.C., Pencharz P.B., De Silva N., Feldman B.M., Fitzgerald P., Sigalet D., Dicken B., Turner J., Marchand V. (2017). Preventing the Progression of Intestinal Failure–Associated Liver Disease in Infants Using a Composite Lipid Emulsion: A Pilot Randomized Controlled Trial of SMOFlipid. J. Parenter. Enter. Nutr..

[12] Nandivada P., Fell G.L., Gura K.M., Puder M. (2016). Lipid emulsions in the treatment and prevention of parenteral nutrition–associated liver disease in infants and children1–3. Am. J. Clin. Nutr..

[13] Olieman J.F., Penning C., Ijsselstijn H., Escher J.C., Joosten K.F., Hulst J.M., Tibboel D. (2010). Enteral Nutrition in Children with Short-Bowel Syndrome: Current Evidence and Recommendations for the Clinician. J. Am. Diet. Assoc..

[14] Olieman J., Kastelijn W. (2020). Nutritional Feeding Strategies in Pediatric Intestinal Failure. Nutrients.

